# Genetic self-counselors in Tunisia: the role of health education in hemoglobinopathies prevention among high school students

**DOI:** 10.1186/s13023-025-03958-z

**Published:** 2025-08-13

**Authors:** Foued Maaoui, Imen Moumni, France Arboix-Calas, Ines Safra, Samia Mnif

**Affiliations:** 1 Laboratory of molecular and cellular hematology, Pasteur Institute of Tunis, Tunis, Tunisia; 2 ISEFC Bardo, Virtual University of Tunis, Tunis, Tunisia; 3 Faculty of Education, University of Montpellier, Montpellier, France

## Abstract

**Background:**

In Tunisia, the primary prevention of hemoglobinopathies relies on behavioral changes related to screening and genetic counseling. The progression in cognitive and functional literacy in human genetics serves as a crucial aspect of this transformation. In this study, we consider the possibility of genetic self-counseling, checking it in students with scientific or literary backgrounds.

**Methods:**

To assess potential for genetic self-counseling applied to sickle cell disease (SCD), we designed a questionnaire on SCD knowledge (SCDKA), then recruited 356 students (200 scientific students vs. 165 literary students). Since and there were no previous standards for classifying students according to their SCDKA score, we considered participants with an SCDKA score ≥ 70% correct answers as having a high literacy level. Statistical analysis of the results was carried out using chi-square tests and Fisher’s, to compare the demographic and educational characteristics of the participants.

**Results:**

The analysis of responses to the various SCDKA items shows a lack of awareness about the hereditary origin of hemoglobinopathies. 97.8% of respondents did not recognize electrophoresis as a diagnostic technique. In terms of genetic literacy, the autosomal and recessive nature of hemoglobinopathies is not well understood. This explains why 41% and 74% of these students could not answer items on genetic transmission. The perception of controllability of hereditary diseases is higher among science students, as is the intention to inform their partner before procreation (56.5% vs. 24.35%, *p* < 0.001). Responses reveal that science section respondents have higher proactive preventive intentions compared to literature section students, as they recognize the usefulness of genetic counseling (75.5% vs. 47.43%, *p* < 0.001), premarital diagnosis (57.5% vs. 18.59%, *p* < 0.001), and prenatal diagnosis (61.5% vs. 13.46%, *p* < 0.001).

**Conclusion:**

Currently, levels of health literacy and functional genetic literacy do not ensure genetic self-counseling for hemoglobinopathies prevention. The survey shows that having a scientific background was an advantage, but a detailed analysis of these students’ results reveals average to low SCDKA scores.

## Introduction

Since the late 1960s, preventive approaches to public health have emphasized risk factor management through behavior modification, integrating socio-cultural contexts with laboratory findings to reduce disease incidence [1, 2]. This model empowers individuals to take an active role in their health, aligning with Michel Foucault’s concept of “practices of the self,” which describes how individuals act upon themselves to manage health risks [3].

In the context of hemoglobinopathies, such as sickle cell disease, primary prevention in Tunisia relies on enhancing health literacy to promote informed decisions about genetic risks. This study explores the potential of genetic self-counseling, enabled through health education, to empower Tunisian high school students to address the risks of hemoglobinopathies within their economic, cultural, and religious contexts.

Tunisia, a North African country in the Mediterranean region, has a population of approximately 11.8 million, with Islam as the predominant religion, practiced by over 99% of the population, primarily Sunni Muslims. Islamic values, including family-oriented traditions and modesty, significantly influence health-related decisions, particularly regarding marriage and reproduction, which are critical for hemoglobinopathies prevention.

Culturally, Tunisia blends Arab, Berber, and Mediterranean influences, with consanguineous marriages being common, increasing the risk of genetic disorders like sickle cell disease and thalassemia [4, 5]. This cultural practice underscores the need for premarital genetic counseling. Economically, Tunisia is classified as a lower-middle-income country, limited healthcare funding and infrastructure, particularly in rural areas, restrict access to genetic testing and counseling services, posing challenges to implementing effective prevention programs.

These socio-cultural and economic factors shape the context for our study, which explores the role of health education in fostering self-genetic counseling to address hemoglobinopathies in Tunisian high schools.

How can we equip citizens with the knowledge and skills to fully play their roles as active health partners and engage them in the face of these hereditary diseases?

Health literacy, defined as the personal, cognitive, and social skills that enable individuals to access, understand, and use health information to promote and maintain good health, is critical for the primary prevention of hemoglobinopathies, such as sickle cell disease (SCD) [6].

In Tunisia, where SCD and other hemoglobinopathies are prevalent due to high consanguinity rates, enhancing health literacy can empower individuals to make informed decisions about genetic risks, including carrier screening and premarital counseling [7].

The World Health Organization emphasizes health promotion through education to reduce the burden of hemoglobinopathies, advocating for early interventions to foster informed health choices [8]. This study investigates how health education in Tunisian high schools can build genetic literacy to support self-counseling, enabling students to act as active health partners in preventing hemoglobinopathies within their socio-cultural and economic contexts.

WHO emphasizes individual empowerment for self-detection disease, including the self-detection of breast cancer [9–10]. These interventions aim to empower individuals to take an active role in their health by providing them with the knowledge and tools needed to detect potential health issues early. The organization highlights the need for health literacy and education to ensure that individuals can effectively use these self-care practices.

These interventions can lead to early detection and better management of various health conditions. We can also consider other examples where individual empowerment is valued, such as self-monitoring of blood glucose for individuals with diabetes [11]. Similarly, self-monitoring of blood pressure at home can help individuals with hypertension manage their condition more effectively and reduce the risk of heart disease and stroke [12]. Another example is self-sampling for Human Papillomavirus (HPV), which involves women collecting their own samples for HPV testing, used to screen for cervical cancer [13].

Why not consider the same autonomy in facing the risks of hemoglobinopathies?

Communication interventions on sickle cell disease and thalassemia already exist [14–17], but are they enough to overcome sustainably the socio-economic, cultural, and religious constraints in African countries?

Since the mapping of the human genome in the 1990s, the ability to diagnose and treat hereditary diseases has steadily improved. As more genetic diseases have been identified, medical centers have been able to utilize genetic testing, and genetic counselors have been hired in more medical subspecialty areas [18–19].

In Africa, the management of genetic diseases faces numerous challenges [20]. Among the obstacles that can hinder the implementation of a primary prevention program for hemoglobinopathies are logistical difficulties, such as public participation from local and/or national health officials; infrastructure limitations, such as appropriate space and equipment along with maintenance plans; and difficulties in procuring consumable materials for screening. Budget constraints often prevent the implementation and sustainability of these programs. Among the major challenges, articles mention the reluctance of families and the community to engage [21–22]. These difficulties also exist in Tunisia. One significant issue is the lack of awareness, both among patients and the public, regarding the availability and benefits of genetic testing and counseling. Additionally, funding gaps and a lack of appropriate legislative frameworks hinder progress in prevention, particularly as policymakers are often unaware of the importance of carrier screening. Furthermore, education on hemoglobinopathies is often absent from school curricula. The social unawareness of these diseases is often responsible for the persistent discrimination against affected individuals [23].

Accessibility is another major concern, exacerbated by workforce shortages and limited healthcare infrastructure. Specialized centers are scarce, financial support for genetic testing is minimal, and there is a lack of African-specific data, despite ongoing research efforts.

Affordability constitutes a further barrier, as many patients cannot afford the high costs of genetic technologies, or the travel required for healthcare. For low- and middle-income social categories, individuals are unable to afford the cost of medical consultations, which hampers preventive interventions based on carrier screening and premarital and prenatal genetic counseling. Finally, the applicability of genetic services in Africa is restricted by the lack of reliable epidemiological data, making it difficult to prioritize services that are locally relevant.

Cultural and religious beliefs also contribute to these challenges, further complicating the integration and efficacity of genetic services into local healthcare systems.

Genetic counseling and premarital screening are essential for identifying at-risk couples and assisting them in making well-informed marriage decisions. Nonetheless, marriage is a complicated topic in Arab-Muslim societies. Couples can still decide to marry even if they are classified as at-risk. The couple and their family may experience severe social stigma or disgrace because of the annulment [24, 25]. Furthermore, even though certain nations provide therapeutic abortion and prenatal diagnosis, couples may decide not to use these services because of cultural and religious constraints. Thus, for successful hemoglobinopathies prevention, increasing health literacy specific to hemoglobinopathies is crucial [26].

Given the deeply ingrained nature of cultural beliefs, attitudes, and perceptions, the cultural environment is an essential component of any educational intervention. These studies [24, 26] demonstrate that the effectiveness of any hemoglobinopathies prevention method may be restricted in the absence of broad-based health education from an early age.

Considering the challenges and obstacles specific to low- and middle-income countries (LMICs), the development of genetic counseling skills from an early age is an interesting option to explore. The aim is not to turn everyone into genetic counselors, but to raise collective awareness of hemoglobinopathies and how to prevent them. It is also about preparing them to play an enlightened role in health and procreation.

In 1975, the American Society of Human Genetics adopted a fundamental definition of genetic counseling. It is a communication process that addresses the human issues associated with the occurrence or risk of occurrence of a genetic disease in a family [27].

This process involves an attempt by one or more duly trained individuals to help the person or family to (a) understand the medical facts, including the diagnosis, the likely course of the disorder, and the available management options; (b) appreciate how heredity contributes to the disorder and the risk of recurrence among specified family members; (c) understand the alternatives for managing the risk of recurrence; (d) choose a course of action that seems appropriate to them, given their risk, family goals, and ethical and religious standards, and act in accordance with that decision; and (e) make the best possible adjustment to the disorder in an affected family member and/or to the risk of recurrence of the disorder.

In 2006, the National Society of Genetic Counselors (NSGC) proposed a more contemporary definition of genetic counseling [28].

Genetic counseling is the process that helps people understand and adapt to the medical, psychological, and familial implications of genetic contributions to disease. This process includes the following elements: (a) interpretation of family and medical histories to assess the risk of disease occurrence or recurrence; (b) education about inheritance, testing, management, prevention, resources, and research; and (c) counseling to promote informed choices and adaptation to the risk or condition.

Based on the scope of practice for genetic counselors approved by the NSGC (2020) [29], the teaching of genomics and human genetics in high school should develop cognitive and psychosocial skills that help learners to: (a) assess individual and family medical histories to determine the genetic risk of hemoglobinopathies in their partner, future children, or relatives; (b) discuss the characteristics, medical history, diagnostic methods, genetic factors, and risk management of diseases; (c) identify medical professionals or counselors to confirm their suspicions and understand the advice from professionals who will recommend genetic laboratory tests; (d) integrate the results of genetic laboratory tests and other diagnostic studies; (e) understand the clinical implications of genetic testing on decisions regarding marriage, reproduction, or abortion; (f) reflect on the anticipatory guidance and options provided by the professional counselor; (g) advocate for certain decisions with their partner, family, or community. Furthermore, the teaching of genetics and genomics in high school should enable learners to reflect on and appropriately respond to ethical and moral dilemmas specific to local contexts.

In Tunisia, genetic counseling is integrated into public health through mandatory premarital counseling programs, established in 1964 and expanded in 1995 [7], to address hereditary diseases such as hemoglobinopathies, particularly in consanguineous populations [30].

Prenatal and postnatal services, including fetal ultrasound screening, are available, though their effectiveness is limited by training and equipment quality [7].

Research by figures like Professor Mongi Ben Hamida has advanced the identification of founder mutations, facilitating cost-effective diagnosis and counseling [7]. However, genetic counseling is not recognized as an independent profession in Tunisia, and services are primarily delivered by physicians or clinical geneticists rather than dedicated genetic counselors [31]. This aligns with broader trends in many African countries, where genetic counseling is often physician-led due to limited training programs and regulatory frameworks [32].

For instance, South Africa, a leader in genetic counseling in Africa, has trained professionals since 1988, with approximately 60–80 individuals practicing after completing specialized training [33, 34]. In contrast, Tunisia lacks formal genetic counseling training programs, and no specific data confirms the presence of master’s-trained genetic counselors [31]. This gap highlights the need for enhanced training and professional recognition to support hemoglobinopathies prevention efforts.

Our study addresses this by exploring the potential of self-genetic counseling and health education among Tunisian high school students to complement existing medical frameworks.

Our goal is not to replace healthcare professionals responsible for genetic counseling with citizens trained in human genetics and hemoglobinopathies. Instead, it is to facilitate mediation between the two. An informed decision in reproductive health can only be made through an understanding of the disease, its genetic transmission, and its physical and psychosocial impacts on patients and their families. Achieving this level of awareness and understanding of procreative options should be built over time, through school education from a young age, rather than through two or three sessions with a professional counselor, regardless of their expertise.

In this study, we investigate whether learning genomics, human genetics and SCD prepares youths to play a committed role in the primary prevention of hemoglobinopathies, to be active partners with healthcare professionals and to develop students’ genetic self-counseling skills.

This investigation among secondary school students aims to assess genetic literacy concerning sickle cell disease (SDC) and its prevention. We answer the following research questions:

- How much do high school students know about SCD?
- Does study specialty influence levels of health literacy and functional genetics literacy applied to SCD?

We hope that the results of this research will guide curriculum designers in creating a competency framework suitable for the primary prevention of SCD.

### SCD education at Tunisian secondary school

Tunisian high school students in the science stream receive information about sickle cell disease during biology classes. The study of genetics in high school, which spans three years, uses this hemoglobinopathy to explain scientific concepts in genomics and human genetics.

For second and third-year science students, the study of genetics uses various teaching aids and begins with an examination of genes and traits, hemoglobin and its function in the body, SCD, and the cause of SCD.

In bachelor’s degree, they receive explanations about the hereditary transmission mode of SCD and the amplifying effect of consanguinity. The biology teacher informs them about prenatal diagnostic techniques and helps them learn how to interpret pedigrees and electrophoresis results. Institutional teaching resources are made available to students during sessions, but the educator is free to use different pedagogies and visual aids to facilitate learning.

The number of sessions, which last about 55 min, is scheduled in advance but can be extended if students have additional questions or if the educator feels that students have not acquired the essential knowledge in genetics. Application exercises and assessments are carried out at the end of the module. For three-quarters of Tunisian high school students who do not belong to the science stream, they receive no education in genetics and SCD as they do not have biology classes.

### Study design, participants, and recruitment

This cross-sectional prospective study was conducted in two public high schools in Tunisia, which offer general curricula including both science and literary streams, as is standard in the Tunisian education system, rather than a specialized focus on sciences. Only 3rd and 4th-year students, aged 16–19, were included, totaling 356 participants (200 from the science stream, with 87 in 3rd year and 113 in 4th year, and 156 from the literary stream).

Science stream students were selected for their exposure to genomics, human genetics, and sickle cell disease (SCD) education in the Tunisian high school biology curriculum, while literary stream students, who do not receive this education, served as a comparison group to assess the impact of genetic education on literacy levels.

The sample size was determined using Slovin’s formula, with a 5% margin of error and 95% confidence level, based on the eligible student population in the two schools, adjusted for logistical constraints and COVID-19 restrictions.

Eligible students, aged 16 to 19, were informed of the objectives of this study and their rights to refuse or stop responding to the survey at any time without any repercussions. Each student was allowed to participate but had to complete the questionnaire independently.

This sample enabled detection of significant differences in SCD knowledge between groups (*p* < 0.001).

### Study procedures

After completing the parental ethical agreement, participants completed the post-education survey independently on a paper at the end of the learning module. The post-education survey included a demographic survey, a health knowledge assessment and the SCT knowledge assessment: SCDKA (Table 1). The same SCDKA questionnaire was distributed to literary stream participants who did not take part in the learning module. Interviewer and biology teacher were present while the students completed the questionnaire. To avoid floor effects, teacher, under investigator’s supervision, was allowed to read questions and explain them to the students.

## Measures

### Health and genetic literacy assessment

This study used SCDKA (Table 1) to assess students’ knowledge of genetic testing and counseling. For the questionnaire design, we drew inspiration from studies aimed at measuring knowledge of healthcare professionals and public about hemoglobinopathies, screening and genetic counseling [35, 36, 37, 38].

Designed to be comprehensible to non-health specialists, we tested the SCDKA with 26 high school science students to ensure its comprehensibility.

In this study, SCDKA also includes questions on health, procreation and SCD transmission, which are covered in human genetics teaching at high school.

Since and there were no previous standards for classifying students according to their SCTKA score, we considered participants with an SCDKA score ≥ 70% correct answers as having a high literacy level. Statistical analysis of the results was carried out using chi-square tests and Fisher’s, to compare the demographic and educational characteristics of the participants.

## Results

During the study period, 356 high school students enrolled in public high schools were invited to participate in the study. Among these students, 200 belonged to a science stream, 87 of whom were in the 3rd year and had basic knowledge of genomics and hemoglobinopathies, and 113 in the 4th year had completed the modules on reproduction and human genetics.

Additionally, 156 students from a literary stream participated in the study without having received any education in genetics (Table 1). The results are recorded in Table 2.

**Table 1:** Students’ self-reported demographic data

Total cohort *n* = 356	Age		Gender		Discipline studied		School level	
	16–17	18–19	Male	Female	Science	Letter	3rd	4th
**Frequency**	166	190	100	256	200	156	172	184
**Percentage**	46.7	53.3	28.1	71.9	56.18	43.82	48.4	51.6

**Table 2:** Sickle cell disease knowledge assessment and percentage of students that answered correctly

SCDKA question and answer choices	Correct *n* (%)	Incorrect *n* (%)	Don’t know *n* (%)	Pearson’s χ2	*P*
1. Can you “catch” Sickle cell disease like the flu? (No)	195 (54.77)	56 (15.73)	104 (29.21)	71.95	<0.001
Life Sciences	147 (73.5)	13 (6.5)	40 (20)		
Letters	48 (31)	43 (27.7)	64 (41.3)		
2. What laboratory test is used to identify a Sickle cell disease?	7 (2)	227 (63.8)	121 (34)		
a. Urine test					
b. Electrophoresis test				50.57	<0.001
c. Blood count					
d. Induced hyperglycemia					
e. Lipid balance					
Life Sciences	6 (3)	154 (77)	40 (20)		
Letters	1 (0.64)	73 (47.09)	81 (52.25)		
3. Can a child carrying a Sickle cell disease allele (heterozygote) develop symptomatic Sickle cell disease? (No)	108 (30.33)	41 (11.51)	207 (58.14)	12.81	0.046
Life Sciences	31 (15.5)	66 (33)	103 (51.5)		
Letters	10 (6.41)	42 (26.92)	104 (66.66)		
4. Can a man with Sickle cell disease avoid having sick children? (Yes)	106 (29.77)	102 (28.65)	148 (41.57)	50.06	<0.001
Life Sciences	87 (43.5)	56 (28)	57 (28.5)		
Letters	19 (12.18)	46 (29.48)	91 (58.33)		
5. It is important to tell your partner about your family Sickle cell disease history, even if you’re healthy. (Yes)	151 (42.41)	128 (35.95)	77 (21.63)	74.54	<0.001
Life Sciences	113 (56.5)	14 (7)	73 (36.5)		
Letters	38 (24.35)	63 (40.4)	55 (35.25)		
6. Could a husband who is heterozygous for Sickle cell disease and his wife who is homozygous for Sickle cell disease have a daughter suffering from the disease? (Yes)	148 (41.57)	24 (6.74)	184 (51.68)	70.47	<0.001
Life Sciences	118 (59)	14 (7)	68 (34)		
Letters	30 (19.23)	10 (6.41)	116 (74.36)		
7. Could a husband with homozygous Sickle cell disease and his healthy (non-carrier) wife have a boy with symptomatic Sickle cell disease? (No)	89 (25)	125 (35.11)	142 (39.88)	87.53	<0.001
Life Sciences	52 (26)	106 (53)	42 (21)		
Letters	37 (23.71)	19 (12.18)	100 (64.10)		
8. Is it wise to carry out premarital genetic counselling if one of the partners is ill? (Yes)	225 (63.2)	58 (16.3)	73 (20.5)	30.95	<0.001
Life Sciences	151 (75.5)	22 (11)	27 (13.5)		
Letters	74 (47.43)	36 (23.07)	46 (29.5)		
9. Is it wise to carry out premarital genetic counseling if the couple is in good health, but the husband’s family has a Sickle cell disease history? (Yes)	144 (40.45)	62 (17.41)	150 (42.13)	80.62	<0.001
Life Sciences	115 (57.5)	41 (20.5)	44 (22)		
Letters	29 (18.59)	21 (13.46)	106 (67.95)		
10. Is it wise to carry out prenatal diagnosis when the couple is healthy, but the woman has Sickle cell disease in her family. (Yes)	144 (40.45)	83 (23.31)	129 (36.23)	88.93	<0.001
Life Sciences	123 (61.5)	36 (18)	41 (20.5)		
Letters	21 (13.46)	47 (30.13)	88 (56.41)		

SCDKA, sickle cell disease knowledge assessment

“I don’t know” was considered an incorrect response

*P* values compare scientific and literary students’ responses

### SCD knowledge and competence among science and letters students

We admit that for an individual to be able to self-counsel in genetics applied to SCD, he or she must be proficient in different forms of literacy: health literacy and genetic literacy.

Health literacy is the ability to access, understand, evaluate, and use health information to make informed decisions and take appropriate actions regarding one’s health [39].

Genetic literacy refers to the ability to understand and make informed decisions about genetic information and its implications for health, reproduction, and personal well-being [40]. Functional genetic literacy refers to the skills needed to apply genetic knowledge in real-life situations, such as making health-related decisions. Its various forms of literacy are largely academic constructs that develop over time in school through biology and health education courses. It is the sum total of scientific knowledge, cognitive and psychosocial skills that equip learners to deal with health risks in their daily lives.

To achieve a good level of knowledge and competency in SCD, we estimate that an SCDKA score of ≥ 70% should be attained. Table 2 shows the percentage of students who answered each SCDKA question, correct response rate for the SCDKA is just 37%.

For the ten items, the comparison of results reveals a significant difference between science students and literature students with a success rate of 47.15% Vs 19.7%, *p* < 0.001.

For most of these young people, their current level of health and genetic literacy does not allow them to self-genetic counseling for anticipating SCD.

#### Health literacy on SCD

SCD is a genetic disorder. It is caused by a mutation in the HBB gene that produces hemoglobin, the protein in red blood cells responsible for carrying oxygen. This mutation causes red blood cells to become rigid and shaped like a sickle, leading to various complications.

The analysis of responses to various items on the SCDKA reveals a lack of awareness regarding the hereditary origin of hemoglobinopathies (item 1: 73.5% vs. 31%, *p* < 0.001).

For 69% of literature students, these diseases are often confused with iron deficiency anemia.

SCD is typically diagnosed through a blood test called hemoglobin electrophoresis, which identifies the presence of sickle hemoglobin. For item 2, (97.8%) of respondents did not recognize electrophoresis as a diagnostic technique.

#### Functional genetic literacy

SCD is an autosomal recessive inheritance pattern, meaning both parents must carry the sickle cell gene for a child to be affected. If both parents are carriers (each having one sickle cell gene and one normal gene), there is a 25% chance with each pregnancy that the child will have SCD, a 50% chance the child will be a carrier, and a 25% chance the child will be unaffected.

#### SCD transmission

In terms of genetic literacy, the autosomal recessive nature of hemoglobinopathies is not well understood (item 3: 15.5% vs. 6.41%, *p* < 0.046). Additionally, 84.5% of science students could not answer the question, “Can a child carrying a thalassemia allele (heterozygote) develop symptomatic thalassemia?” This explains why 41% and 74% of these students could not answer items 6 and 7 on genetic transmission.

Among the options that can help anticipate and avoid the transmission of SCD are Carrier Screening. Before marriage or during pregnancy, individuals can undergo screening tests to determine if they are carriers of the sickle cell gene. This can help make informed reproductive decisions. There is also Preimplantation Genetic Diagnosis (PGD).

For those undergoing in vitro fertilization (IVF), PGD can be used to test embryos for SCD before implantation. This ensures that only unaffected embryos are implanted.

We asked our students if they were aware of these options and if they accepted them.

#### SCD controllability

The perception of the controllability of hereditary diseases is higher among science students (item 4: 43.5% vs. 12.18%, *p* < 0.001), as is the intention to inform their partner before procreation (item 5: 56.5% vs. 24.35%, *p* < 0.001).

Responses to items 8, 9, and 10 show that respondents from the science section have higher proactive preventive intentions compared to literature section students. They recognize the usefulness of genetic counseling (item 8: 75.5% vs. 47.43%, *p* < 0.001), premarital diagnosis (item 9: 57.5% vs. 18.59%, *p* < 0.001), and prenatal diagnosis (item 10: 61.5% vs. 13.46%, *p* < 0.001).

## Discussion

Hemoglobinopathies, including sickle cell disease (SCD), pose a significant health and economic burden in Tunisia, exacerbated by high consanguinity rates [7]. Our findings indicate that current levels of health and genetic literacy among Tunisian high school students are insufficient for effective self-counseling to prevent hemoglobinopathies.

To address this, integrating SCD education into school curricula and health center programs, as successfully implemented in Bahrain [41], could enhance literacy and promote preventive behaviors, such as carrier screening and genetic counseling. Such interventions should align with local socio-cultural practices to ensure acceptance and effectiveness, supporting WHO’s call for health promotion to reduce hemoglobinopathy incidence [8, 42].

The World Health Organization (2022) report “Health literacy development for the prevention and control of non-communicable diseases (NCDs)” [43] provides a relevant framework for literacy development to improve prevention and control of NCDs. It calls for an approach that involves practitioners, organizations, health systems, and policymakers meaningfully engaging with and supporting communities to create and sustain supportive health environments based on local needs and within the framework of social practices and resources.

For health literacy to have an impact on the prevention and management of hemoglobinopathies in Africa, it is necessary to understand how it can be used by individuals in their daily lives and whether it fits the particular contexts of African countries and LMICs.

How do you go from being a “passive citizen” to a “committed partner”?

We believe that it is possible and necessary to become a self-counsellor in SCD, which implies having sustainable knowledge and skills in human genetics.

Sickle cell disease (SCD) was selected as the focus of this study due to its inclusion in the Tunisian high school biology curriculum as a key example for teaching genomics and human genetics, making it an effective model for assessing genetic literacy among students. Its autosomal recessive inheritance pattern and well-documented clinical implications facilitate evaluating students’ understanding of genetic transmission and preventive strategies, such as carrier screening and genetic counseling.

In Tunisia, SCD is a significant public health concern, with a carrier frequency for the sickle cell trait estimated at 1.89% nationally and up to 12.5% in high-risk regions, driven by high consanguinity rates (25–30% of marriages) and historical malaria endemicity [4, 7].

Prenatal diagnosis data (1994–2012) indicated that 40.3% of 461 fetuses from at-risk couples were at risk for SCD, underscoring its prevalence among hemoglobinopathies [7]. Given this burden, we recommend integrating education on SCD genetics, transmission, and prevention into elementary and high school basic science curricula to enhance genetic literacy and promote early awareness.

Additionally, incorporating genetic counseling services in health centers, as successfully implemented in Bahrain [41], could support premarital screening and reduce SCD incidence. These interventions align with WHO recommendations for reducing hemoglobinopathy burden through education and counseling and could empower Tunisian communities to mitigate the impact of SCD.

### Genetic self-counselling, not for everyone

In this study, we investigate whether learning genomics, human genetics and SCD prepares youths to play a committed role in the primary prevention of hemoglobinopathies, to be active partners with healthcare professionals and to develop students’ genetic self-counseling skills.

Given our results, the current biology curriculum does not provide learners with the level of knowledge and engagement needed to anticipate hereditary transmission risks.

Functional literacy in genetics involves the ability to evaluate risky premarital and reproductive situations. Learning this skill requires knowledge of human genetics, which only one-third of Tunisian secondary school students possess.

The survey shows that having a scientific background was an advantage, but a detailed analysis of these students’ results reveals average to low SCTKA scores. Scientific students have a good basic literacy in genetics. However, their functional and critical literacies in genetics and health remain weak. They struggled to solve situations related to thalassemia, as they did not know it is an autosomal recessive disease.

In many nations, including Bangladesh and Saudi Arabia [44], students with a science background scored highest on knowledge assessment for thalassemia, while those with an arts and humanities background scored lowest [45]. Numerous studies stress the value of health education in helping students embrace preventive actions [44, 45, [46].

Being in a literary stream seems to disadvantage many Tunisian students, as confirmed by our survey results, where 50% of participants reported being unable to assess the risk of having children with thalassemia. Genetic concepts are not only complex but also linguistically challenging for secondary school students [47]. The technical vocabulary is extensive, filled with multisyllabic terms like chromosome, heterozygote, homozygote, or polymerase, and terms specific to genetic entities and phenomena that do not relate to an adolescent’s daily life, such as allele, meiosis, mitosis, etc [48, 49] These words’ meanings cannot be inferred from everyday experiences [50]. 

However, proficiency in scientific language, essential for understanding scientific culture and its main concepts, has been recognized as crucial since Vygotski’s research (1978) and the rise of socioconstructivism in education [51]. 

Literary students found it extremely difficult to respond to our SCTKA questionnaire, which is understandable given the lack of genetic education in secondary school.

This educational disparity should concern education authorities. How can one be an informed participant, a health partner, and a committed citizen regarding hemoglobinopathies without a basic understanding of genetics? How can we engage in societal debates on genetic issues?

Given the complexity of hemoglobinopathies, health education cannot be confined to a single discipline. Health education should not be limited to one category of students, as the health issues and citizenship prospects are common to all.

### Towards curricular reform in favor of genetic self-counseling

The perspective of empowerment towards hemoglobin disorders must be supported by cognitive justice in the scientific learning of human genetics in schools. The knowledge of these diseases, their physical and psychosocial repercussions, prevention, and therapy measures require an adequate didactic and pedagogical approach.

It must be part of the health promotion framework and in partnership with all stakeholders. This education of youth must be complemented during adulthood with effective health communication on the prevention and management of hemoglobinopathies.

This strategy must be inclusive and avoid epistemic injustice, i.e. “.the structural exclusion of marginalized producers and recipients of knowledge” [52].

In the health field, epistemic injustice refers to public health interventions that are not based on what a society knows or how it perceives the world [52].

Countries and regions have their own complex systems of influence on health and health behaviors through historical, geographic, political, environmental, sociocultural, traditional, ancestral, religious, legal, and economic factors.

Health literacy development should be based on an understanding of the social practices, contexts and value systems in which people are born, grow, live, work and age [53, 54].

For primary prevention of hemoglobinopathies, health literacy can be part of an individualistic or community-based approach. Programs, interventions, and activities should focus on building knowledge of human genetics and changing individual and community behavioral and emotional attitudes toward early diagnosis and genetic counseling.

Furthermore, these programs must be compatible with the worldview and health aspirations of an African citizen. They must not conflict directly with religious or ethical beliefs or oppose directly important social practices. Therefore, there is a need to take a holistic approach to health literacy by integrating contextual factors into health education or communication interventions.

As children grow and develop, they learn about prevailing social practices, influences, and traditions, including taboos, related to health [55]. The biology curriculum should therefore aim at acquiring knowledge in genetics as well as developing cognitive and psychosocial skills that guide learners in making informed health decisions.

### Limits

For both feasibility and Covid-19 restrictions, the sample was limited to two public schools.

A larger-scale longitudinal survey could confirm our findings.

In addition, our results are based on declarative data collected by teachers and are therefore subject to social desirability bias.

## Conclusion

At present, levels of health literacy and functional genetic literacy do not ensure genetic self-counseling for hemoglobinopathies prevention. Nevertheless, improvement is possible provided that the biology curriculum taught in high schools is reformed. It is up to policymakers to subscribe hemoglobinopathy education a priority on a par with communicable diseases.

This study demonstrates that Tunisian high school students, particularly those in literary streams, exhibit limited health and genetic literacy regarding sickle cell disease (SCD), hindering their ability to engage in self-counseling for hemoglobinopathies prevention.

Our findings underscore the critical role of health education in equipping students with the knowledge and skills to understand SCD’s genetic transmission and preventive measures, such as carrier screening and premarital counseling. By fostering genetic literacy, students can act as informed health partners, supporting self-help and assisting community members in making informed health decisions within Tunisia’s socio-cultural context.

We recommend integrating SCD genetics and prevention education into high school science curricula. While a national program for SCD screening, diagnosis, and treatment could further reduce disease burden, our study primarily advocates for educational interventions to empower young people as genetic self-counselors, laying the foundation for sustainable prevention strategies.

## Data Availability

The datasets used and/or analyzed during the current study are available from the corresponding author on reasonable request.
